# Elucidating the Diversity of Aquatic *Microdochium* and *Trichoderma* Species and Their Activity against the Fish Pathogen *Saprolegnia diclina*

**DOI:** 10.3390/ijms17010140

**Published:** 2016-01-21

**Authors:** Yiying Liu, Christin Zachow, Jos M. Raaijmakers, Irene de Bruijn

**Affiliations:** 1 Department of Microbial Ecology, Netherlands Institute of Ecology (NIOO-KNAW), Wageningen 6708 PB, The Netherlands; y.liu@nioo.knaw.nl (Y.L.); j.raaijmakers@nioo.knaw.nl (J.M.R.); 2 Laboratory of Phytopathology, Wageningen University, Wageningen 6708 PB, The Netherlands; 3 Austrian Centre of Industrial Biotechnology (ACIB GmbH), Graz 8010, Austria; christin.zachow@acib.at; 4 Institute of Biology, Leiden University, Leiden 2333 BE, The Netherlands

**Keywords:** salmon, Saprolegniosis, *Microdochium*, *Trichoderma*

## Abstract

Animals and plants are increasingly threatened by emerging fungal and oomycete diseases. Amongst oomycetes, *Saprolegnia* species cause population declines in aquatic animals, especially fish and amphibians, resulting in significant perturbation in biodiversity, ecological balance and food security. Due to the prohibition of several chemical control agents, novel sustainable measures are required to control *Saprolegnia* infections in aquaculture. Previously, fungal community analysis by terminal restriction fragment length polymorphism (T-RFLP) revealed that the Ascomycota, specifically the genus *Microdochium*, was an abundant fungal phylum associated with salmon eggs from a commercial fish farm. Here, phylogenetic analyses showed that most fungal isolates obtained from salmon eggs were closely related to *Microdochium lycopodinum/Microdochium phragmitis* and *Trichoderma viride* species. Phylogenetic and quantitative PCR analyses showed both a quantitative and qualitative difference in *Trichoderma* population between diseased and healthy salmon eggs, which was not the case for the *Microdochium* population. *In vitro* antagonistic activity of the fungi against *Saprolegnia diclina* was isolate-dependent; for most *Trichoderma* isolates, the typical mycoparasitic coiling around and/or formation of papilla-like structures on *S. diclina* hyphae were observed. These results suggest that among the fungal community associated with salmon eggs, *Trichoderma* species may play a role in *Saprolegnia* suppression in aquaculture.

## 1. Introduction

Saprolegniosis, caused by *Saprolegnia* species, results in tremendous losses in wild and cultured fish species including salmonids such as salmon and trout, and non-salmonids such as tilapia, catfish, carp, and eel [1]. The typical symptoms of Saprolegniosis are white or grey fungal-like hyphal mats on fish or their eggs [2]. Yield losses of 10% to more than 50% have been reported in eggs and young fish [1,3,4].

To control Saprolegniosis, formalin is now commonly applied but is expected to be banned soon due to adverse effects on the environment [1]. A limited number of chemical and non-chemical alternative treatments have been tested to control Saprolegniosis, including hydrogen peroxide, sea water flushes and ultraviolet irradiation, but none of these are as effective as the banned malachite green [1]. Also, no vaccine is currently available to control this disease [1,5].

Bacterial genera such as *Bacillus*, *Enterococcus* and *Lactobacillus* have been shown to reduce specific diseases in aquaculture and several of these beneficial bacteria are being commercialized [6,7,8,9,10]. As a sustainable measure to combat Saprolegniosis, the bacterial genera *Aeromonas*, *Frondihabitans* and *Pseudomonas* have been proposed [1,11,12,13,14,15,16,17,18]. Alike the probiotic bacteria, several beneficial fungi and/or their bioactive compounds are applied to control diseases. These fungal species are isolated either randomly or systematically [19,20,21,22,23,24,25]. Amongst commercialized fungi, *Aspergillus oryzae*, *Coniothyrium minitans*, *Phlebiopsis gigantea* and *Trichoderma* (teleomorph *Hypocrea* [26]) spp., are able to suppress diseases and promote the growth of various hosts, mainly terrestrial crops and some animals such as cattle [27,28,29,30]. For fish, *Trichoderma viride* enhanced body weight and reduced mortality of Nile tilapia exposed to *Saprolegnia* sp. [31]. The commercial product HetroNex, containing *Trichoderma viride* and *Trichoderma harzianum*, has been developed to control fungal and oomycete diseases caused by *Fusarium*, *Lagenidium* and *Saprolegnia* in aquaculture ponds of fish, prawn and shrimp [32].

In a fungal diversity study of the marine sponge *Dragmacidon reticulatum*, *Trichoderma* represented one of the most abundant genera among the isolated fungi [33]. To date, however, still little is known about the fungal community in aquaculture or aquatic environments [18,34]. Previously, we showed by clone library analyses that the oomycete community associated with *Saprolegnia*-infected (diseased) and healthy salmon eggs from a commercial fish hatchery were dominated by *Saprolegnia* with no difference in the number and pathogenicity of the *Saprolegnia* isolates present in either diseased or healthy salmon egg batches [18]. Based on terminal restriction fragment length polymorphism (T-RFLP) analysis and clone library sequencing, also no obvious differences were observed in the fungal community composition between the diseased and healthy salmon egg batches [18]. The clone library consisted of 209 fungal clones, the majority of which belonged to the Ascomycota. More specifically, 139 clones were classified as *Microdochium* (teleomorph *Monographella*) [18,35,36]. To elucidate the role of fungi in the protection of salmon eggs against Saprolegniosis, we isolated and (phylogenetically) characterized fungi from diseased and healthy salmon eggs. Their abundance in diseased and healthy salmon egg batches and their activity against *Saprolegnia diclina* were investigated here.

**Figure 1:**
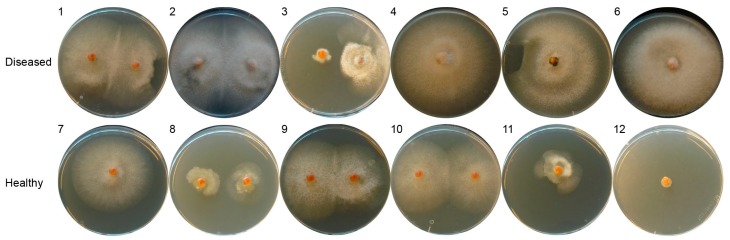
Isolation of salmon egg-associated fungi and oomycetes on potato dextrose agar plates. One or two salmon eggs from a *Saprolegnia*-infected batch (diseased, replicate No. 1–6) or a healthy batch (replicate No. 7–12) were placed onto the agar plates to allow fungal outgrowth.

## 2. Results and Discussion

### 2.1. Isolation of Fungi from Diseased and Healthy Salmon Eggs

Previously, *Saprolegnia*-infected (diseased) and healthy salmon eggs and their corresponding incubation water were sampled from a commercial fish farm (*N* = 6 for diseased eggs and *N* = 6 for healthy eggs) [18]. Per sample, one or two salmon eggs were placed on potato dextrose agar to allow fungal outgrowth (Figure 1). We obtained and purified 20 fungal isolates in total and by ITS sequencing identified three different genera, *Microdochium*, *Trichoderma* (both Ascomycota) and *Mortierella* (Zygomycota). *Microdochium* and *Mortierella* were the most represented genera in our previous clone library sequence analysis. Interestingly, *Trichoderma* was not detected in the previous analysis, probably due to the limited number of sequenced clones, the specificity of the primers or the efficiency of the PCR reaction [18]. The two *Mortierella* isolates (BLAST identity of 100% in Genbank database) were isolated from only one healthy salmon egg sample. Here we aimed at comparing isolates obtained from multiple replicate samples of diseased and healthy eggs. Therefore, we focused on the Ascomycota isolates for the subsequent analyses described below.

### 2.2. Microdochium

**Figure 2:**
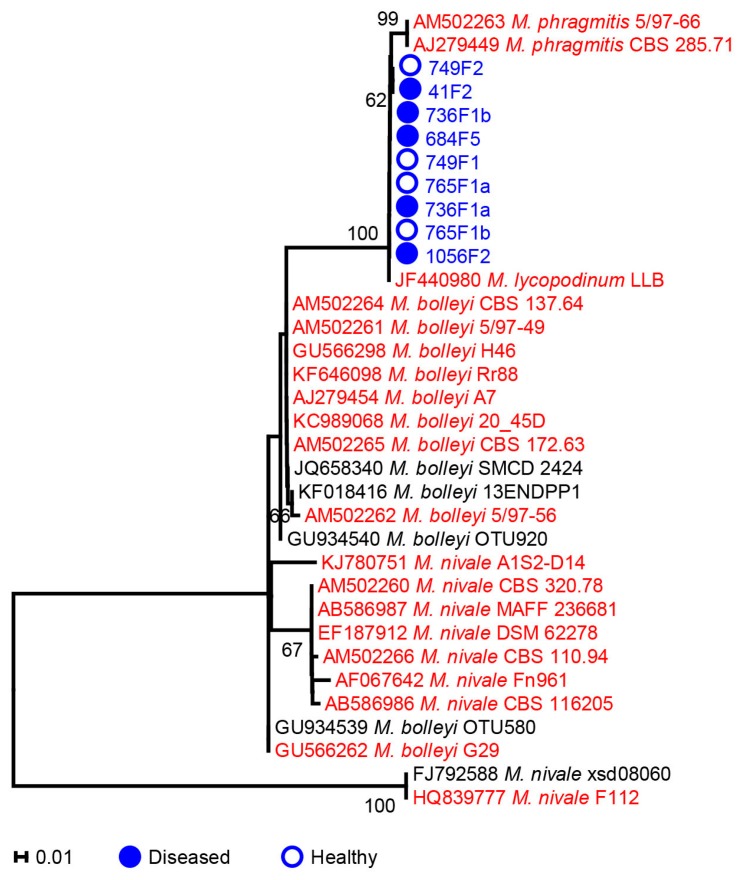
Phylogenetic tree of ITS rRNA sequences of nine *Microdochium* isolates from salmon eggs and reference strains. The phylogenetic analyses were conducted in Mega 5 using the Kimura 2-parameter method [48] to compute evolutionary distances. The bootstrap values indicated at the nodes are based on 1000 bootstrap replicates. Branch values lower than 50% are hidden. Closed and open circles indicate *Microdochium* isolates from *Saprolegnia*-infected (diseased) or healthy salmon egg samples, respectively. Red, blue and black colors indicate strains from terrestrial/plant, aquatic and unknown sources of isolation, respectively. The scale bar indicates an evolutionary distance of 0.01 nucleotide substitution per sequence position. Twenty-five ITS sequences of good quality and at least 550 bp of reference strains of *Microdochium* were downloaded from GenBank; their strain names are preceded by the accession numbers.

**Figure 3:**
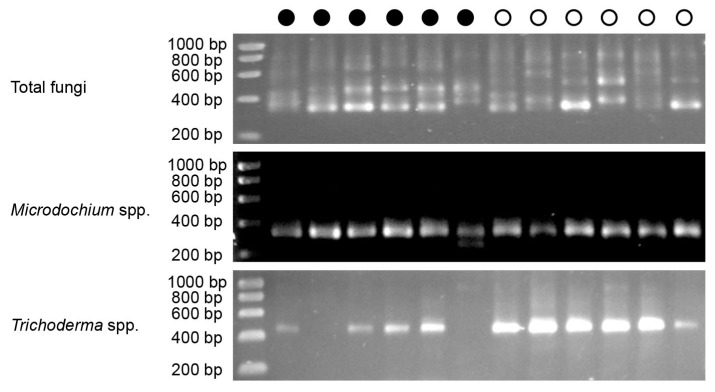
Detection of total fungi, *Microdochium* and *Trichoderma* in salmon egg samples by quantitative PCR. Total fungal community was detected using ITS4-ITS9 primers, *Microdochium lycopodinum/Microdochium phragmitis* species were detected by MPF-MPR primers, and *Trichoderma* species were detected by ITS1TrF-ITS4TrR primers. The concentration of DNA template of each sample was normalized at 5 ± 1 ng·μL^−1^. Closed and open circles indicate DNA samples extracted from *Saprolegnia*-infected (diseased) or healthy samples, respectively. The lane on the left is the size marker and the band size (bp) is indicated next to each band.

**Figure 4:**
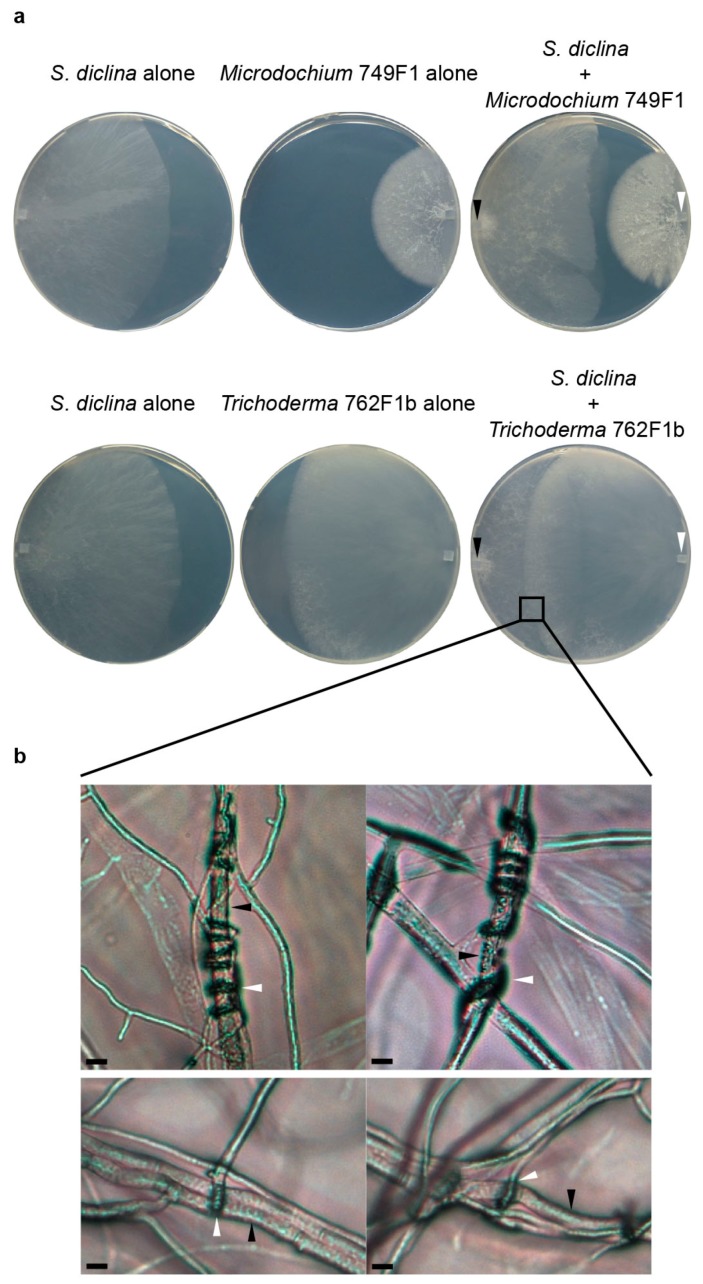
*In vitro* activities of *Microdochium* and *Trichoderma* isolates. (**a**) Dual culture of *Microdochium* isolate 749F1 or *Trichoderma* isolate 762F1b with *Saprolegnia diclina* 1152F4 on 1/5th strength potato dextrose agar (1/5PDA). *S. diclina* and the fungal isolates were pre-grown on 1/5PDA. A hyphal plug of *S. diclina* was inoculated on the left side of the fresh 1/5PDA and a hyphal plug of *Microdochium* isolate 749F1 or *Trichoderma* isolate 762F1b was inoculated on the right side. The dual cultures were incubated for six days at 20–25 °C. The black arrows indicate *S. diclina* plugs and the white arrows indicate *Microdochium* or *Trichoderma* plugs; (**b**) Microscopic pictures of the hyphal interaction between *Trichoderma* and *S. diclina*. The black arrows indicate hyphae of *S. diclina*. The white arrows indicate the coiling of *Trichoderma* hyphae around *S. diclina* hyphae (**top** pictures) or the formation of papilla-like structure of *Trichoderma* hyphae around *S. diclina* hyphae (**bottom** pictures). Scale bars represent 10 μm.

*Microdochium* species are known as snow molds, and some are pathogenic to plants [37,38,39,40,41,42,43,44]. *Microdochium nivale* and *Microdochium majus* are two of the main causative agents of *Fusarium* head blight [42], whereas *Microdochium lycopodinum* and *Microdochium* *phragmitis* were isolated from plants without causing disease [45,46]. *M.* *phragmitis* was endophytic in common reed and was more present in flooded habitats than the closely related *Microdochium bolleyi* [45]. Some *Microdochium* species were antagonistic to the plant pathogen *Verticillium dahliae* [47]. Amongst our nine *Microdochium* isolates, five were isolated from diseased and four from healthy salmon eggs (Table 1). The origin of our *Microdochium* isolates was possibly from the catchment area, which was the water source for the salmon egg incubators [18]. Based on the phylogenetic analyses of internal transcribed spacer (ITS) sequences, all the nine *Microdochium* isolates are closely related to *M. lycopodinum* and *M. phragmitis*, and no distinct separation is observed between isolates from diseased or healthy salmon eggs (Figure 2). Quantitative PCR using *M. lycopodinum*/*M. phragmitis* specific primers showed and confirmed that *M. lycopodinum*/*M. phragmitis* was detected in equal amounts in total DNA samples obtained from diseased and healthy salmon egg samples (Figure 3). One *Microdochium* isolate (749F1) inhibited the hyphal growth of *Saprolegnia diclina* on 1/5th strength potato dextrose agar (1/5PDA) (Table 1, Figure 4a) suggesting the secretion of enzymes or other bioactive metabolites.

**Table 1:** *In vitro* activity of *Microdochium* or *Trichoderma* isolates against hyphal growth of *Saprolegnia diclina* 1152F4. The fungal isolates were retrieved from *Saprolegnia*-infected (diseased) or healthy salmon egg samples.

Genus	Strain No.	Salmon Egg Sample	Activity of Culture Filtrate	Dual Culture Assay on 1/5PDA	Hyphal Interaction with *S. diclina* Microscopically
*Microdochium*	41F2	Diseased	Not inhibitory	Not inhibitory	Not observed
	684F5	Diseased	Not inhibitory	Not inhibitory	Not observed
	736F1a	Diseased	Not inhibitory	Not inhibitory	Not observed
	736F1b	Diseased	Not inhibitory	Not inhibitory	Not observed
	1056F2	Diseased	Not inhibitory	Not inhibitory	Not observed
	765F1a	Healthy	Not inhibitory	Not inhibitory	Not observed
	765F1b	Healthy	Not inhibitory	Not inhibitory	Not observed
	749F1	Healthy	Not inhibitory	Inhibitory	Not observed
	749F2	Healthy	Not inhibitory	Not inhibitory	Not observed
*Trichoderma*	684F1	Diseased	Not inhibitory	Not inhibitory	Coiling, papilla-like structure
	1056F1	Diseased	Not inhibitory	Not inhibitory	Papilla-like structure
	1152F1	Diseased	Not inhibitory	Not inhibitory	Inconclusive
	762F1a	Healthy	Not inhibitory	Not inhibitory	Coiling
	762F1b	Healthy	Not inhibitory	Not inhibitory	Coiling
	762F2	Healthy	Not inhibitory	Not inhibitory	Papilla-like structure
	764F1	Healthy	Inhibitory	Not inhibitory	Papilla-like structure
	764F2	Healthy	Not inhibitory	Not inhibitory	Coiling, papilla-like structure
	764F3	Healthy	Not inhibitory	Not inhibitory	Coiling, papilla-like structure

To date, not much is known about *Microdochium* in aquatic environments, aquaculture or aquatic animals [18]. Also not much is known about the bioactive compounds produced by *Microdochium*. Bhosale *et al.* (2011) reported that the active compound cyclosporine A, extracted from an estuarine *M. nivale*, has the potential to be applied pharmaceutically to control diseases caused by some dermatophytes and *Aspergillus* species in human and animals [49]; Santiago *et al.* (2012) reported that the extract of *M. phragmitis*, which was isolated from Antarctic angiosperms, showed cytotoxic activity against a human tumoral cell line [50]. Therefore, further experiments are needed to decipher the bioactive potential capacity of our *Microdochium* isolates, especially their interaction with pathogens from cold water environments, like *Saprolegnia* spp.

### 2.3. Trichoderma

Most *Trichoderma* species are applied in agriculture as biocontrol agents against various plant-associated bacterial, fungal and oomycete pathogens, such as *Clavibacter*, *Fusarium* and *Phytophthora* [26,51,52]. *Trichoderma* species are capable of producing a range of extracellular compounds to suppress plant pathogens, such as enzymes, fungicidal compounds and antibiotics; they can also promote plant growth via symbiotic association with plant hosts [26,51,53,54,55]. *Trichoderma* is commonly isolated from terrestrial environments, such as soil and wood [26], but also from aquatic environments like freshwater (drinking water) and marine water [33,56,57,58,59,60]. Marine *Trichoderma atroviride* and *Trichoderma asperelloides* suppressed disease caused by *Rhizoctonia solani* on beans and enhanced defence responses against pathogenic *Pseudomonas syringae* pv. Lachrimans on cucumber seedlings [57]. Some other marine-derived *Trichoderma* strains were capable of producing antagonistic compounds against cancer, diabetes, cancer cell lines or pathogenic *Staphylococcus epidermidis*; such compounds include tandyukisins from *Trichoderma* *harzianum* OUPS-111D-4, pyridones from *Trichoderma* sp. MF106, and trichoketides from *Trichoderma* sp. TPU1237 [58,61,62].

**Figure 5:**
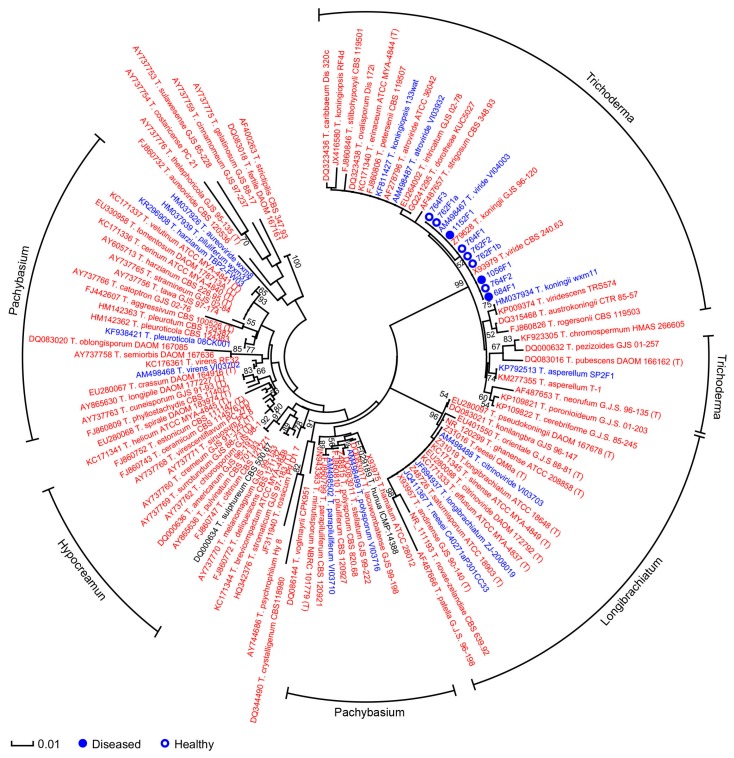
Phylogenetic tree of ITS rRNA sequences of nine *Trichoderma* isolates and reference strains. The phylogenetic analyses were conducted in Mega 5 using the Tamura 3-parameter method [70] to compute evolutionary distances. The bootstrap values indicated at the nodes are based on 1000 bootstrap replicates. Branch values lower than 50% are hidden. Closed and open circles indicate isolates from *Saprolegnia*-infected (diseased) or healthy samples, respectively. Red, blue and black colors indicate strains from terrestrial/plant, aquatic and unknown sources of isolation, respectively. The scale bar indicates an evolutionary distance of 0.01 nucleotide substitution per sequence position. Outer labels describe section names based on the list of species in ISTH website [64] and only sections contained at least five strains are indicated. 104 ITS sequences of good quality and at least 550 bp of reference strains of *Trichoderma* were downloaded from GenBank; their corresponding strain names are preceded by the accession numbers. Strain names followed by “(T)” indicate type strains.

**Figure 6:**
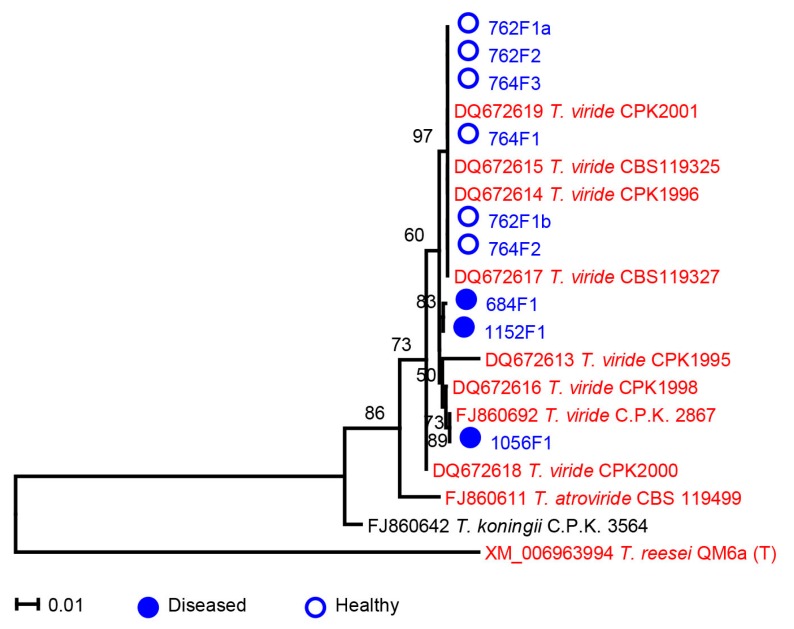
Phylogenetic tree of *tef1* sequences of nine *Trichoderma viride* isolates and reference strains. The phylogenetic analyses were conducted in Mega 5 using the Tamura-Nei method [71] to compute evolutionary distances. The bootstrap values indicated at the nodes are based on 1000 bootstrap replicates. Branch values lower than 50% are hidden. Closed and open circles indicate isolates from *Saprolegnia*-infected (diseased) or healthy samples, respectively. Red, blue and black colors indicate strains from terrestrial/plant, aquatic and unknown sources of isolation, respectively. The scale bar indicates an evolutionary distance of 0.01 nucleotide substitution per sequence position. 11 sequences of good quality and at least 1200 bp of reference strains of *Trichoderma* were downloaded from GenBank; their strain names are preceded by the accession numbers. Strain name followed by “(T)” indicates type strain.

It was suggested that *Trichoderma* may have the potential to also control infectious diseases in aquaculture [63]. Among our nine *Trichoderma* isolates, three were isolated from diseased and six from healthy salmon eggs (Table 1). Quantitative PCR with *Trichoderma*-specific primers showed that *Trichoderma* was present in higher abundance in total DNA samples of healthy than of diseased salmon eggs (Figure 3), although some variation in results were observed between replicated PCR reactions on the same DNA samples (Supplementary Material, Figure S1). The total fungal community did not differ in abundance between healthy and diseased salmon eggs (Figure 3). Collectively, these results suggest that *Trichoderma* is more enriched in healthy salmon egg samples than in diseased salmon egg samples. Phylogenetic analyses based on ITS sequences showed that all nine *Trichoderma* isolates belonged to the *Trichoderma* section [64] and no apparent separation was observed between isolates from diseased or healthy salmon eggs based on ITS sequences (Figure 5). However, phylogeny based on sequences of the translation elongation factor 1 alpha (*tef1*) clearly separated the *Trichoderma* isolates from diseased and healthy salmon eggs (Figure 6). Our nine *Trichoderma* isolates and the *Trichoderma viride* reference strains [65,66] formed three clades of *Trichoderma viride*. These results suggest that next to a quantitative difference also a qualitative difference in *Trichoderma* populations from diseased and healthy salmon eggs.

In terms of extracellular activity, we observed that the culture filtrate of only one *Trichoderma* isolate showed inhibition of hyphal growth of *S. diclina* (Table 1). Dual culture assays did not show inhibition of hyphal growth of *S. diclina* by any of the *Trichoderma* isolates tested (Table 1, Figure 4a). However, the hyphae of most *Trichoderma* isolates coiled around or produced a papilla-like structure on the hyphae of *S. diclina* (Table 1 and Figure 4b) [26]. The coiling and papilla-like structures suggest attachment of *Trichoderma* hyphae to *S. diclina* hyphae. Coiling is required for mycoparasitism but not all coiling leads to mycoparasitism [26,54,55,67]. The formation of papilla-like structures in the interaction with *S. diclina* could indicate the start of mycoparasitic invasion by *Trichoderma*; these structures have been shown to induce hyphal breakdown of various hosts [26,54,55,68,69].

Even though *Trichoderma* species are commonly considered beneficial fungi, some *Trichoderma* strains, including *T. harzianum*, *Trichoderma koningii*, *Trichoderma longibrachiatum*, *Trichoderma pseudokoningii* and *Trichoderma viride*, maybe pathogenic to human [72,73,74,75,76,77,78,79,80,81,82,83,84,85,86,87]. Some marine *Trichoderma* were associated to contaminated mussels and some were even toxic to aquatic animals, such as *Artemia* larvae [25,88]. Therefore, evaluations of the adverse effects of our *Trichoderma* isolates on the environment and humans are needed.

Previous work by Abdelhamid *et al.* (2007) indicated that *T. viride* can enhance body weight and reduce mortality of Nile tilapia treated with *Saprolegnia* sp. [31]. Our isolates belong to the *T. viride* clade and are, to our knowledge, the first characterized *Trichoderma* from salmon eggs. Collectively, our results pointed to both quantitative and qualitative differences in *Trichoderma* population between diseased and healthy salmon eggs. These analyses suggest a potential role of *Trichoderma* species in the protection of salmon eggs from *S. diclina*. Hence, our *Trichoderma* isolates and/or their metabolites, especially isolate 764F1 and its bioactive compounds, may have the potential to be applied in aquaculture. To this end, *in vivo* experiments should be conducted to determine the beneficial effects of our *Trichoderma* isolates in controlling Saprolegniosis and other aquaculture diseases.

## 3. Experimental Section

### 3.1. Phylogenetic Analysis of Microdochium and Trichoderma Isolates

Fungal isolation from salmon eggs was described previously by Liu *et al.* [18]. DNA isolation, internal transcribed spacer (ITS) rRNA sequencing and phylogenetic analyses of fungal isolates were conducted as described by Liu *et al.* [18]. For *Trichoderma* isolates, additional phylogenetic analysis was conducted with translation elongation factor 1 alpha (*tef1*) sequences. The *tef1* gene was amplified by primer set EF1-728F [89] and TEF1LLErev [90]. The evolutionary distances of the phylogenetic trees were computed using the Kimura 2-parameter method [48] for *Microdochium* ITS sequences, Tamura 3-parameter method [70] for *Trichoderma* ITS sequences and Tamura-Nei method [71] for *tef1* sequences.

### 3.2. Culture Filtrate Activity of Microdochium and Trichoderma Isolates

One agar plug of each *Microdochium* and *Trichoderma* isolate was pregrown in 6 mL 1/5th strength potato dextrose broth (1/5PDB, Difco™, Franklin Lakes, NJ, USA) for one week at 20–25 °C. Each culture was lyophilized, the pellet dissolved in 6 mL fresh 1/5PDB and filter-sterilized through a 0.2 μm filter (Whatman™, Freiburg, Germany). 1 mL culture filtrate solution was added into a well of 24 well suspension culture plate (Greiner bio-one, Cellstar^®^, Frickenhausen, Germany) and one agar plug of *Saprolegnia diclina* 1152F4 was added into each well. After incubation at 14–15 °C for four days the effect of the culture filtrates on hyphal growth of *S. diclina* was determined.

### 3.3. Dual Culture Assay

One agar plug of each of the *Microdochium* or *Trichoderma* isolates and one agar plug of *S. diclina* were placed at two opposite sides of 1/5th strength potato dextrose agar (1/5PDA). After incubation for 6 days at 20–25 °C, inhibition of hyphal growth of *S. diclina* was determined and plates were stored for one to two months at 4 °C until microscopic analyses were performed. Hyphal interactions were observed under a Nikon 90i epifluorescence microscope (Nikon Instruments Europe BV, Amsterdam, The Netherlands) with brightfield settings and accomplished with Nikon NIS-elements.

### 3.4. Quantification of Total Fungi, Microdochium and Trichoderma in Salmon Egg Incubation Water

DNA extraction from salmon egg samples and storage was described in Liu *et al.* [18]. All DNA samples were thawed on ice and normalized to 5 ± 1 ng·μL^−1^. To quantify total fungi in the water samples, ITS rRNA genes were amplified with ITS4 [91] and ITS9 [92] primers in 12 μL volumes, each consisted of 5 μL of DNA template, 5.8 μL BIOLINE 2x SensiFAST SYBR No-ROX mix, 0.1 μL ITS4 primer (10 mM), 0.1 μL ITS9 primer (10 mM) and 1 μL BSA (0.1 mg·mL^−1^). The quantitative PCR program consisted of 1 cycle at 95 °C for 5 min, 45 cycles at 95 °C for 20 s, 55 °C for 20 s, 72 °C for 30 s, 82 °C for 15 s. To quantify *Microdochium* *lycopodinum/Microdochium* *phragmitis* in the water samples, MPF (5′-AAGGTACCCGAAAGGGTGCTGG-3′) and MPR (5′-GAATTACTGCGCTCAGAGTACGT-3′) primers were designed and firstly the accuracy was verified by PCR using the genomic DNA isolated from *M. phragmitis* CBS 285.71 and *Microdochium nivale* var. *nivale* CBS 110.94 as template (Supplementary Material, Figure S2). The quantitative PCR program consisted of 1 cycle at 95 °C for 5 min, 45 cycles at 95 °C for 20 s, 60 °C for 20 s, 72 °C for 30 s, 82 °C for 15 s. To quantify *Trichoderma* in the water samples, *Trichoderma* specific genes were amplified with ITS1TrF and ITS4TrR primers [93] and QIAGEN Rotor-Gene^®^ SYBR^®^ Green PCR Master Mix 2x was used. The quantitative PCR program consisted of 1 cycle at 95 °C for 5 min, 45 cycles at 95 °C for 10 s, 51 °C for 10 s, 72 °C for 20 s, 82 °C for 10 s. Genomic DNA of *Trichoderma* isolates 1152F1 and 762F1b, and *Microdochium* isolates 736F1a and 749F1 was isolated with PowerSoil^®^ DNA isolation kit (MO BIO Laboratories, Inc., Carlsbad, CA, USA) according to the manufacturer’s instructions. Dilution series of DNA of each strain was prepared at 5, 5 × 10^−1^, 5 × 10^−2^, 5 × 10^−3^, 5 × 10^−4^, 5 × 10^−5^, 5 × 10^−6^, 5 × 10^−7^ ng·μL^−1^ and used as standards (Supplementary Material, Figure S3).

### 3.5. Nucleotide Sequence Accession Numbers

All DNA sequences have been deposited in GenBank. The accession numbers for the internal transcribed spacer sequences of *Trichoderma*, *Microdochium* and *Mortierella* are KU202214-22, KU202223-31 and KU202232-33, respectively. The accession numbers for the sequences of translation elongation factor 1 alpha of *Trichoderma* isolates are KU202234-42.

## 4. Conclusions

Aquaculture has become one of the fastest developing animal food sectors [94], partly due to regulations to protect wild fish populations from overfishing and the increased demand for fish products [1]. To support this increase in food demand, aquaculture production is gradually intensifying, but effective and sustainable strategies are needed to suppress emerging diseases including Saprolegniosis. Very few studies have demonstrated the beneficial activity of fungi against aquatic pathogens [31,95]. Our study is, to our knowledge, the first to establish correlations between the frequency/occurrence of indigenous fungal communities (*Trichoderma* and *Microdochium* species) and the health status of salmon eggs in a commercial hatchery. Our study is also the first to assess the diversity among *Trichoderma* and *Microdochium* isolates from aquaculture samples. The traditional plate assays provided informative results showing the potential antagonistic activity of our *Trichoderma* isolates obtained from salmon eggs against the pathogen *Saprolegnia diclina*. These results demonstrated the basic characters of our *Trichoderma* isolates, which provide a good starting point for future analyses on the molecular basis of *Trichoderma*-*Saprolegnia* interactions. Further *in vitro* and *in vivo* tests are needed to confirm their beneficial protective activity *in situ*. The role of *Trichoderma* in *Saprolegnia* disease suppression is especially interesting, since *Trichoderma* was shown to be more abundant in healthy salmon eggs than in diseased ones and showed a mycoparasitic interaction with *Saprolegnia*. Our study provides a framework to isolate and monitor putative protective fungi in *Saprolegnia* control and possibly other emerging diseases in aquaculture.

## Supplementary Materials

Supplementary materials can be found at http://www.mdpi.com/1422-0067/17/1/140/s1.
