# Supplementary material for: Elucidating the Diversity of Aquatic Microdochium and Trichoderma Species and Their Activity against the Fish Pathogen Saprolegnia diclina
Source: Int J Mol Sci. 2016 Jan 21;17(1):140. doi: 10.3390/ijms17010140 (PMC4730379; doi:10.3390/ijms17010140)
Supplement: Supplementary file 1 [file ijms-17-00140-s001.pdf]

Supplementary Materials: Elucidating the Diversity of Aquatic *Microdochium* and *Trichoderma* Species and Their Activity against the Fish Pathogen *Saprolegnia diclina*

Yiying Liu, Christin Zachow, Jos M. Raaijmakers and Irene de Bruijn

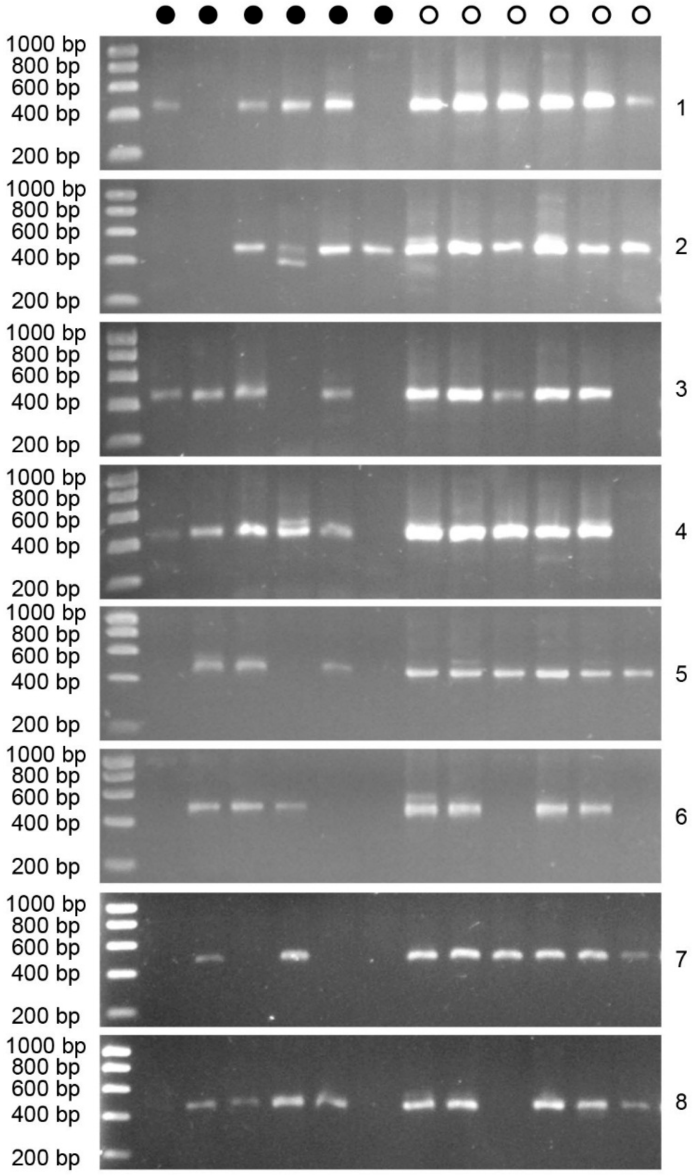

Figure S1. Reproducibility of quantitative PCR based detection of *Trichoderma* species in salmon egg samples. Eight independent quantitative PCRs were run for each salmon egg sample to show the variation of amplification. The concentration of DNA template of each sample was normalized at 5 ± 1 ng·μL^{-1}. Closed and open circles indicate *Saprolegnia*-infected (diseased) or healthy salmon egg samples, respectively. The expected band size is around 500 bp.

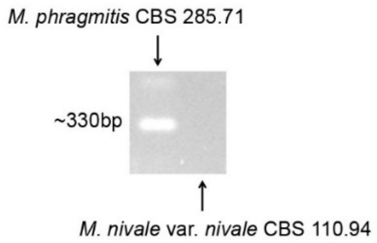

Figure S2. Amplification of *Microdochium lycopodinum*/*Microdochium phragmitis* specific gene by primer set MPF-MPR using *M. phragmitis* CBS 285.71 as positive control and *M. nivale* var. *nivale* CBS 110.94 as negative control.

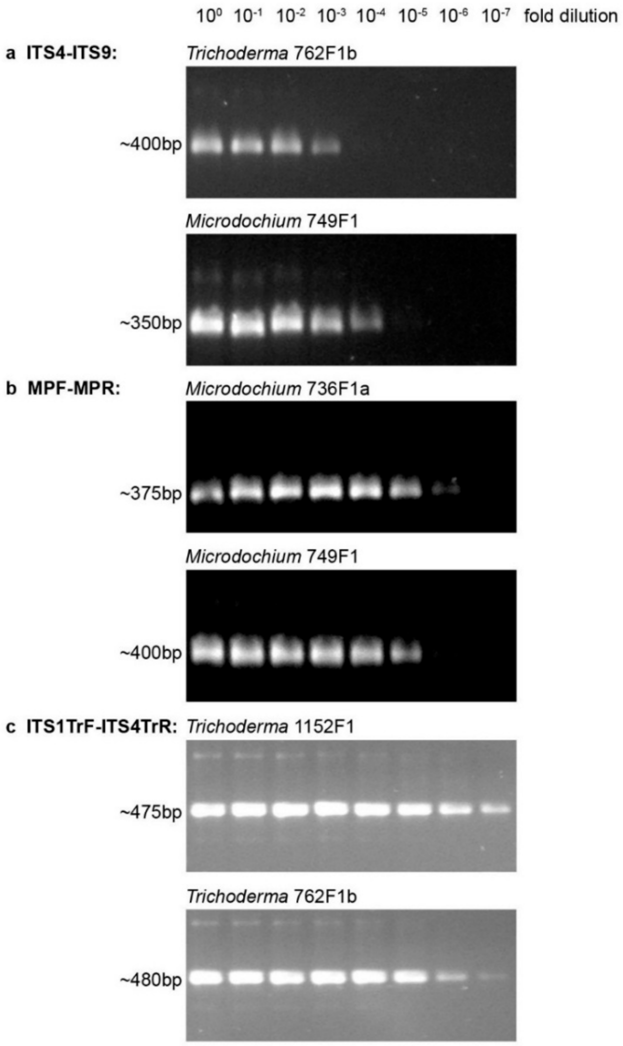

Figure S3. Standards of the quantitative PCR to determine the detection limits. The genomic DNA of the indicated fungal strains was set at 5 ng·μL^{-1} and diluted in 10-fold steps. Each dilution, indicated at the top, was used as template for the PCR using the primer sets ITS4-ITS9 (**a**), MPF-MPR (**b**) and ITS1TrF-ITS4TrR (**c**).
